# *CTEN* induces epithelial-mesenchymal transition (EMT) and metastasis in non small cell lung cancer cells

**DOI:** 10.1371/journal.pone.0198823

**Published:** 2018-07-09

**Authors:** Xiangdong Lu, Juan Gao, Yao Zhang, Tao Zhao, Hongchuan Cai, Tingrong Zhang

**Affiliations:** 1 Department of Oncology, The Jiangyin Hospital Affiliated to Medical College of Southeast University, Jiangyin, P.R.China; 2 Department of Obstetrics and Gynecology, Jiangyin Hospital of Traditional Chinese Medicine, Jiangyin, P.R.China; 3 The 515^th^ Hospital of PLA, Jiangyin, P.R.China; University of South Alabama Mitchell Cancer Institute, UNITED STATES

## Abstract

To explore the effects and mechanism of *CTEN* (COOH-terminus tensin-like molecule) on EMT, cell migration and invasion of Human lung adenocarcinoma cells. The pCMV-vector, pCMV-CTEN, Control-shRNA, and *CTEN*-shRNA were transfected into A549 and NCI-H1299 cells by Lipofectamine 2000. Transforming growth factor-*β1*(TGF-*β1*)and epithelial-mesenchymal transition (EMT) -related biomarkers were detected by eliseand western blot. The migration and invasion ability of A549 cells and NCI-H1299 were examined by scratch-wound assay and transwell assay respectively. We found compare with control group, the expression of *TGF-β* and mesenchymal markers in *CTEN* overexpression group were increased, and the epithelial marker was decreased, which induced the EMT process. Meanwhile, scratch-woundassay showed that the migration efficiency of A549 and NCI-H1299 cells in *CTEN* overexpression group were higher than that in control group.Transwell assay demonstrated that the number of cells that migrated and invaded through the membrane were obviously more than those in control group.Furthermore, Knockdown of *CTEN* partially reversed transforming growth factor-β1(*TGF-β1*)-induced changes in EMT markers. In conclusion, *CTEN* activated the expression of *TGF-β1*, thereby prompting EMT in lung adenocareinma cancer cells.

## Introduction

Lung cancer is the leading cause of death and mortality in malignant tumors worldwide [1]. Non small cell lung cancer (NSCLC) is the most common type of lung cancer which is accounted for about 85% of all lung cancer patients[2]. The occurrence of NSCLC is a complex process involving multiple genes which contains proto-oncogene activation, tumor-suppressor gene inactivation and mutations in many genes[3]. *CTEN* (COOH-terminus tensin-like molecule), which was cloned in 2002, located on chromosome 17q21 region. Its cDNA contains 4015bp encoding a 715 amino acid protein. There are 6 potential tryosine phosphorylation sites in *CTEN* [4] which is involved in cell adhesion, migration and signal transduction[5].*CTEN* was not detected in normal tissues of small intestine, colon, liver and spleen. However, *CTEN* highly expresses in tumors except prostate cancer and is considered as one of the novel oncogenes involved in tumorigenesis[6,7]. *β*

*TGF- β* signaling pathway is closely involved in EMT process. *TGF- β1* is the most representative member of *TGF- β* protein family which contains *TGF- β1*,*TGF- β2* and *TGF- β3*. *TGF- β1* promotes the process of tumor development, invasion and metastasis. Our previous immunohistochemistry (IHC) results showed that the expression levels of *CTEN* and *TGF- β1* were significantly correlated with tumor size, histological grade TNM staging and lymph node metastasis in non small cell lung cancer tissues. And there’s a serious posibility that *CTEN* and *TGF- β1* play important roles in tumorigenesis in NSCLC. But the underlying mechanism is still unknown. In the present study, the expression and potential mechanism of *CTEN* in the development of lung cancer was investigated. To the best of our knowledge, for the frst time it was demonstrated *CTEN* may transcriptionally activate the expression of *TGF- β*, thereby prompting the EMT process of lung cancer cells.

## Materials and methods

### Cell lines and reagents

Human lung adenocarcinoma A549 and NCI-H1299 cell was phurchased from Shanghai Institute of Biochemistry and Cell Biology. Antibodies against *CTEN*, *TGF-β1*, *E-cadherin*, *N-cadherin*, *Vimentin*, *α-smoothmuscle a*ctin (*α-SMA*) and decapentaplegic homolog 2 (*Smad2*) were purchased from Cell Signaling Technology Inc. p-decapentaplegic homolog 2(*p-Smad2*) was purchased from Abcam Inc. Lipofectamine 2000, Trizol and reverse-transcription kit were purchased Invitrogen Inc. Transwell chamber and matrigel were purchased from BD Inc. Puromycin, ECL chemiluminescence staining solution, and PVDF membrane were phuchased from Sigma Inc. RPMI-1640 medium was phuchased from Gibco Inc.

### Cell culture and transfections

A549 cells and NCI-H1299 were grown in RPMI-1640 containing 10% fetal bovine serum at 37°C in a 5% CO_2_ incubator. 5 × 10^5^cells were seeded on each well of 6-well plates the day before transfection. Gene expression constructs were transfected into cells using Lipofectamine 2000 according to the manufacturer’ s instructions. After 36 h transfection, cells were harvested for total RNA and whole cell lysates extraction.

### Real-time PCR

Total RNA was extracted using TriZol reagent. Reverse transcription was used a Quantscript RT Kit. Real time PCR performed using a EvaGreen qPCR Master Mix kit. Primers used were in Table 1.

**Table 1:** Quantitative PCR primer sequence.

Primer name	Primer sequence(5 ' - 3')
*CTEN*	F:ACTGATGTCCAGAGGAAGGTG
	R:ATGTCATACTCCGCAAAGAGG
*E-cadherin*	F: GACCGAGAGAGTTTCCCTACG
	R:TCAGGCACCTGACCCTTGTA
*N-cadherin*	F:GAGATCCTACTGGACGGTTCG
	R:TCTTGGCGAATGATCTTAGGA
*Vimentin*	F:CCTTGAACGCAAAGTGGAATC
	R:TGAGGTCAGGCTTGGAAACAT
*TGF-β1*	F: TCTCCAGGCATTTCCACTATTC
	R: CTCAGGCATTCGTCAACATCTA
*GAPDH*	F: GGTCTCCTCTGACTTCAACA
	R: AGCCAAATTCGTTGTCATAC

### Western blotting

Total cell lysatewas extracted with RIPA buffer.The cell lysate wasresolved in SDS-PAGE and transferred onto PVDF membrane. Then blocked with 5% BSA milk in TBST (TBS with 0.05% Tween 20) and sequentially incubated with primary antibodies and horseradish peroxidase-conjugated secondary antibodies in 5% BSA in TBST. Blots were washed with PBST after each incubation for 1 hour. The immunoreactive bands were visualized by Amersham Biosciences ECL reagents following the provided instructions.

### ELISA dosage

For ELISA experiments, 30 000 cells/cm^2^ were seeded into 24 plates. Cells were lysed by following the manufacturer’s instructions. All samples were stored at -20°C. Briefly, *TGF- β1* were measured using a sandwich ELISA technique according to the manufacturer’s instructions. Detection assay is based on the horseradish peroxidase colorimetric reaction by adding TMB substrate. Absorbance was read at 450 nm immediately.

### In-viro scratch-wound assay

A549 and NCI-H1299 cells were seeded on 6-well plate. The original wounds were inflicted by dragging a sterile 200 μl pipettte tip across the monolayer. Cells within the wound area were washed twice with PBS. Three photomicrographs of each scratch were obtained at the initial time of wound creation and the location was photographed 24 h later. Image analysis software (ImageJ, National Institutes of Health, Bethesda, MD, USA) was used to quantify (in pixels) the area of the wound remaining. This number was then converted to a percentage of the scratch area remaining at each time point.

### In-viro cell invasion assay

The in vitro invasive abilities of A549 and NCI-H1299 cells were evaluated using a transwell chamber coated with 100 μl Matrigel. A total of 2 × 10^4^ cells in 200μl RPMI-1640 medium were introduced into the upper chamber, and 800 μl RPMI-1640 medium with 20% FBS was introduced into the lower chamber. Cells were allowed to invade the Matrigel for 24 h. The invaded cells were fixed by methanol and stained with 0.1% crystal violet. The number of invaded cells was counted under a phase contrast microscope. Cells in five different fields of each well were averaged.

### Short-Hairpin RNA Knockdown of *CTEN* Expression

A549 and NCI-H1299 cells were plated in 6-well culture plates with standard medium for 24 hours. The medium was removed and replaced with medium containing pLKO.1 puromycinresistant lentiviral vectors containing a short-hairpin (sh)RNA sequence targeting *CTEN* or a nontargeted shRNA used as a control. Polybrene (Sigma-Aldrich) was also added at 8*μ*g/mL to facilitate transfection. After 24 hours of exposure to the lentiviral constructs, the medium was replaced with standard medium with puromycin (Sigma-Aldrich) at a concentration of 2 *μ*g/mL to kill any nontransfected cells. Lentiviral was purchased from Shanghai GenePharmCo., Ltd. (Shanghai, China); The shRNA sequence used for *CTEN* knockdown was CCGGCCTTGACTCCTACATTGACTTCTCGAGAAGTCAATGTAGGAGTCAAGGTTTTTTG, which is a validated sequence from Shanghai GenePharmCo., Ltd. (Shanghai, China).

### Statistical analysis

Data are presented as the mean ± standard deviation following 3 independent experiments. Statistical analysis was performed using SPSS 17.0 software. Two-sided *p* values were calculated, and a difference was considered statistically significant if *p*< 0.05.

## Results

### *CTEN* regulates EMT, migration and invasion of A549 and NCI-H1299 cells

**Fig 1:**
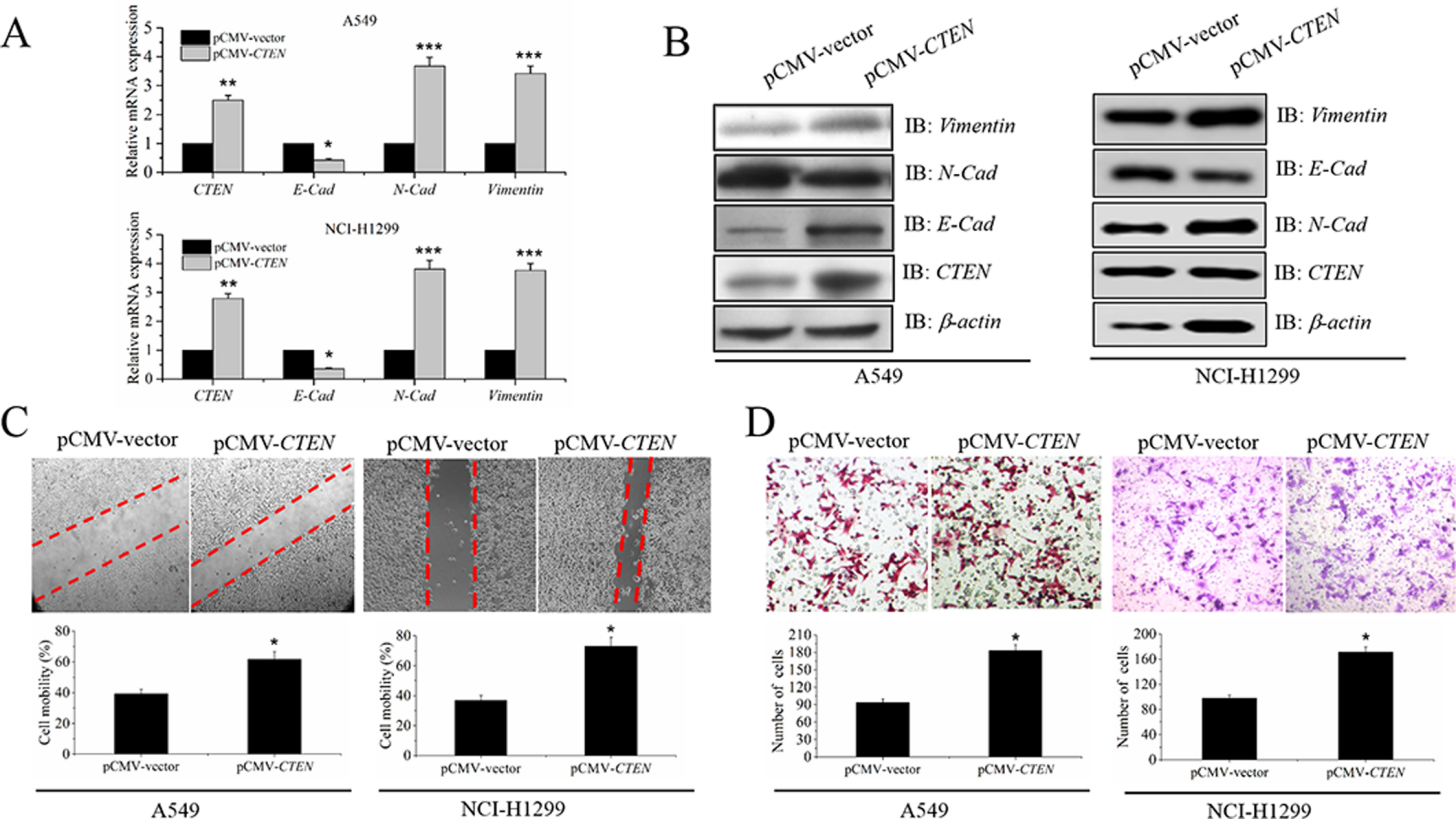
*CTEN* regulates EMT, migration and invasion of A549 and NCI-H1299 cells. *CTEN* positively regulates EMT, migration and invasion of human lung adenocarcinoma A549 and NCI-H1299 cells pCMV-*CTEN* and pCMV-vector control were transfected into A549 and NCI-H1299 cells respectively. Expression level of *CTEN*, *E-cadherin (E-cad)*, *N-cadherin (N-Cad)* and *Vimentin* was examined by real-time PCR (A) and western blotting (B) migration (C) and invasion (D). Representative images depicting the effect of *CTEN* overexpression on A549 and NCI-H1299 cells. P<0.05, **P<0.01, vs.Vector.

To address whether *CTEN* is involved in EMT, migration and invasion of human lung adenocarcinomacell-line A549 and NCI-H1299, we first transfected pCMV-*CTEN* and pCMV-vector control into A549 and NCI-H1299 cells respectively. Then, we examined the expression of *N-cadherin*, *E-cadherin* and *Vimentinin* in the transfected cells. The results showed that the expression of N-cadherin and Vimentin were increased in pCMV-*CTEN* panel than in pCMV-vector control panel by real-time PCR, while the level of E-cadherin was decreased in pCMV-CTEN panel than in pCMV-vector control panel (Fig 1A).These differences can be further confirmed in the protein level by western blotting with *β-actin* as a loading control(Fig 1B). The following *in-viro* scratch-wound assay in A549 and NCI-H1299 cells showed that *CTEN* overexpressed group migrated faster than vector control group (Fig 1C). Furthermore, transwell assay demonstrated that overexpression of *CTEN* enhanced the invasion ability of A549 and NCI-H1299 cells (Fig 1D). Overall, the above results indicate that *CTEN* is a positive regulator of EMT, cell migration and invasion in A549 and NCI-H1299 cells.

### *CTEN* stimulates the expression of *TGF -β1* in A549 and NCI-H1299 cells.

**Fig 2:**
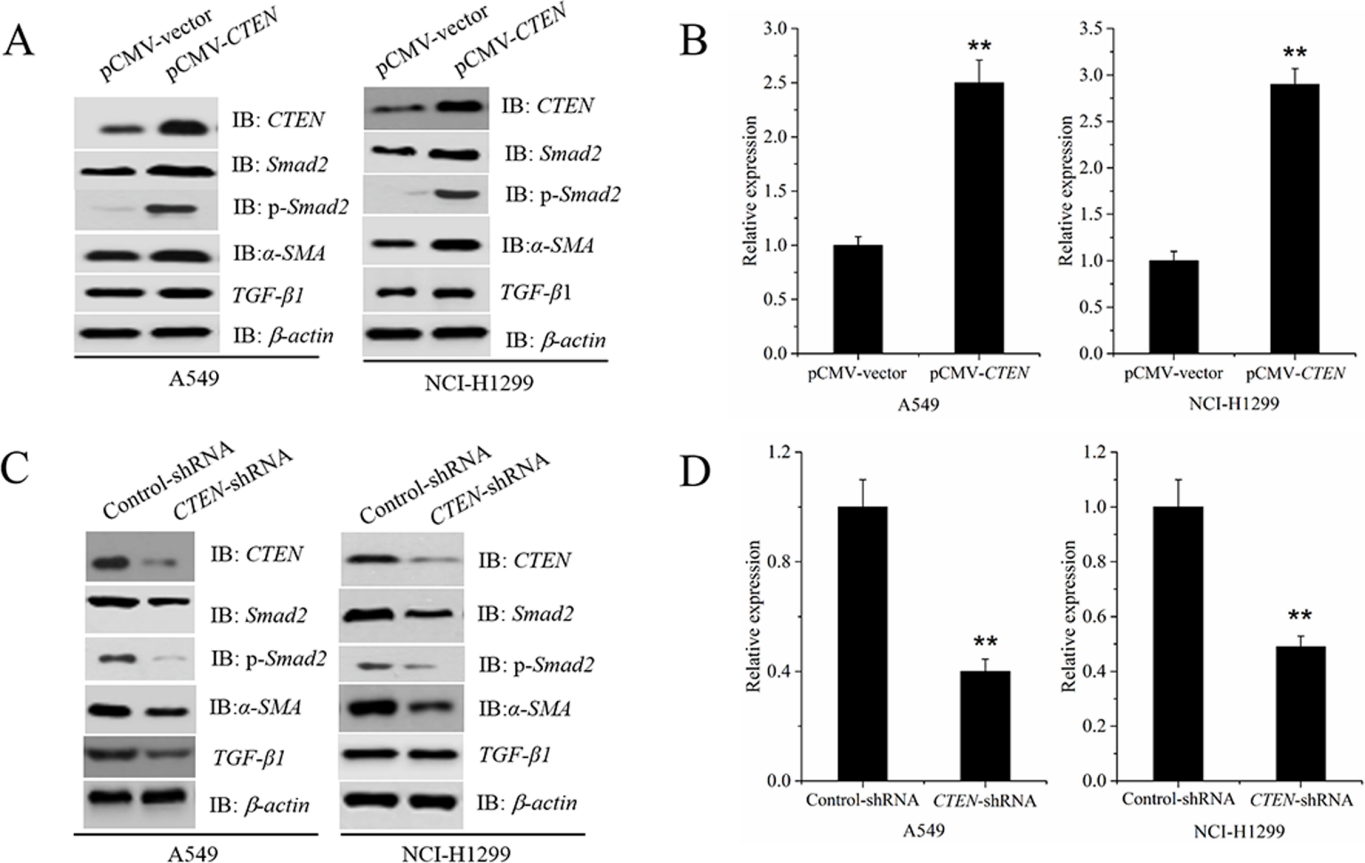
*CTEN* stimulates the expression of *TGF-β1* in A549 and NCI-H1299 cells. Western blot and Eliseanalysis showing that overexpression of *CTEN* signifcantly enhanced the expression *TGF-β1* and the downstream signaling pathway(A-B). Silencing of *CTEN* signifcantly suppressed the expression of *TGF-β1* as well as the downstream effectors *Smad2*, p-*Smad2* and *α-SMA* (C-D). *P<0.05, **P<0.01, vs. Control.

Our study found that *TGF -β1* promotes EMT, migration and invasion of human lung adenocarcinoma A549 cells (S1 Fig). Therefore, we want to study the relationship between *CTEN* and *TGF-β1* in lung cancer cells. Next, pCMV-CTEN and pCMV-vector was transfected into A549 and NCI-H1299 cells for 48 h. Western blot and Elise analysis revealed that overexpression of CTEN significantly enhanced the expression *TGF-β1* (Fig 2A and 2B). And expression of downstream effectors *Smad2*,*p-Smad2* and *α-SMA* were also significantly upregulated (Fig 2A). By contrast, silencing of *CTEN* with a specifc shRNA signifcantly suppressed the protein expression of *TGF-β1*, as well as the downstream effectors *Smad2*, *p-smad2* and *α-SMA* compared with the NC shRNA (Fig 2C). These data indicated that *CTEN* stimulates the expression of *TGF-β1* inducing downstream signaling in A549 and NCI-H1299 cells. The results were verified by Elise (Fig 2C and 2D).

### Silence of *CTEN* partially abolishes *TGF -β1* -induced EMT process in A549 and NCI-H1299 cells

**Fig 3:**
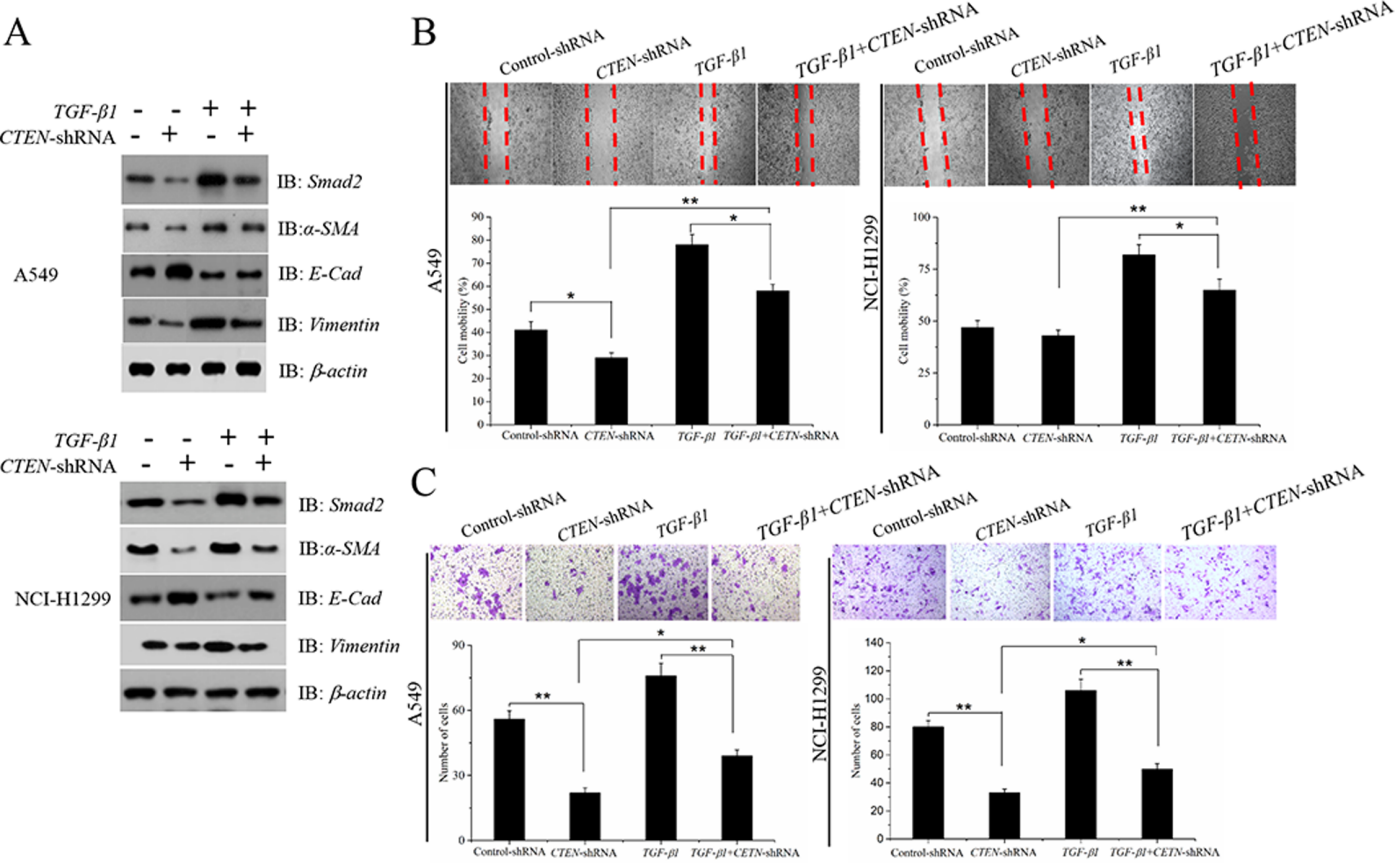
Silence of *CTEN* partially abolishes *TGF-β1*-induced EMT process in A549 and NCI-H1299 cells. Western blot analysis of the *TGF-β1* signaling pathway when A549 and NCI-H1299 cells were treated with sh-*CTEN* and/or *TGF-β1* (A)Scratch-wound changes of A549 and NCI-H1299 cells were determined in cells were treated with sh-*CTEN* and/or TGF-*β1* (B). The invasion of A549 and NCI-H1299 cells were determined in cells were treated with sh-*CTEN* and/or *TGF-β1* (C).*P<0.05, **P<0.01, vs. Control.

To determine whether *CTEN* prompts the EMT process in A549 and NCI-H1299 cells by stimulating *TGF-β1* expression, A549 and NCI-H1299 cells with *CTEN*-shRNA, *TGF-β1*, either alone ortogether. Silencing of *CTEN* signifcantly suppressed the *TGF—β1* signaling pathway. By comparison, treatment knockdown of *CTEN* with *TGF—β1* markedly activated the TGF-β1 signaling pathway, including upregulation of, *Smad2*, *α-SMA*, *Vimentinin* and downregulation of *E-cadherin* (Fig 3A). Notably, *CTEN* partially reversed *TGF-β1* treatment-induced changes to the expression of EMT markers (Fig 3A). The scratch-wound assay of A549 and NCI-H1299 cells were also determined((Fig 3B). As presented in Fig 3C *TGF-β1* induced cell invasion was partially reversed by knockdown of *CTEN*.These data indicated that *CTEN* prompted A549 and NCI-H1299 migration, invasion and EMT, primarily by stimulating the expression of *TGF-β1*. This result suggested that *TGF-β1* was a potential downstream target of *CTEN* in human lung adenocarcinoma.

## Discussion

Lung cancer is the highest incidence and mortality malignant tumor in China[8].Currently, Surgical treatment is the main treatment for lung cancer, combined with chemotherapy, radiotherapy, immunotherapy and other means. In recent years, although great progress has been made in lung cancer, the 5-year survival rate of lung cancer patients is still less than 18%[9].The main reason is the failure to detect, diagnose and treat early. About 85% of lung cancer patients have metastases at the initial diagnosis, and are at an advanced stage with poor prognosis.

*CTEN* is a member of tensin family proteins which plays important role in mediating cell morphology, migration and signal transduction. It was reported that loss of *CTEN* led to prostate cancer[10]. Sasaki H *et al* detected the expression of *CTEN* in 89 cases of lung cancer patients and found that mRNA expression level of *CTEN* was positively correlated with T grade [11], this found indicated that *CTEN* play a role in the progression of lung cancer. In invasive breast cancer, *CTEN*, associated with *EGFR* and *HER2*, contributed to the metastasis of breast cancer cells [7]. *CTEN* enhances transcriptional activity of *STAT3* for enhancing the invasion and metastasis of breast cancer cells. [12]. *CTEN* can increase *EMT* in rectal cancer for reduce the *E-cadherin* level and promote the invasion and metastasis efficiency of rectal cancer cells [7].

EMT, which was firstly proposed by Greenburg and Hay in 1982 [13], refers to the transformation of epithelial cells into stromal cells under specific physiological and pathological conditions. EMT is one of the important factors for tumor invasion and metastasis. The hallmarks of EMT include: The morphological changes of cells from cobblestone to spindle shape;cells lose polarity, and cytoskeletal rearrangement occurs;cells break through the basement membrane to gain athletic capacity and enhanced cellular matrix metalloproteinases (*MMPs*) activity-*MMP2* and *MMP9*; enhanced expression level of stromal cell markers *(N-Cadherin*, *Vimentin* and *Fibronectin*) andreduced expression level of epithelial cell marker (E-Cadherin) [14]. Transforming growth factor (*TGF*), a class of cytokines, plays important role during mammalian embryonic development including regulating cell migration and proliferation, and tissue repair[15]. In recent years,the role of *TGF-β1* in tumorigenesis is attracting more and more attention. It has been reported that *TGF-β1* can induce EMT in many epithelial cells including mammary epithelial cells, liver cells and kidney proximal tubules [16]. And in vitro cell culture and in vivo metastasis experiments also confirm that *TGF-β1* regulates the occurrence of EMT.

Our previous IHC results demonstrated that the expression levels of *CTEN* and *TGF- β1* are both significantly correlated with NSCLC tumor size, histological grade TNM staging and lymph node metastasis. *CTEN* and *TGF- β1* may play important role in tumorigenesis in NSCLC. But the underlying mechanism is still unknown. In the present study, we demonstrated that overexpression of *CTEN* promoted migration and invasion of human lung adenocarcinoma A549 and NCI-H1299 cells. Real-time PCR and Western blotting restults showed that *CTEN* upregulated *N-cadherin* and *Vimentin* level while downregulated *E-cadherin* level. Taken together, these results indicated that *CTEN* can promote the occurrence of EMT of A549 and NCI-H1299 cells and thus elevat migration and invasion of human lung adenocarcinoma.

## Conclusions

Our studies also show that overexpression of *CTEN* promotes *TGF- β1* expression level in A549 and NCI-H1299 cells, and the promotion of *CTEN* on EMT, cell migration and invasion was obviously weakened if we knockdown *TGF- β1* before *CTEN* overexpression. This resuts indicate that the enhancement of *CTEN* on EMT, cell migration and invasion of human lung adenocarcinoma A549 and NCI-H1299 cells is through *TGF- β1*. Thus, *CTEN* is hopefully to be a therapeutic target for invasion and metastasis of non-small cell lung cancer.

## Supporting information

S1 Fig *TGF- β1* promotes EMT, migration and invasion of human lung adenocarcinoma A549 cells. *TGF- β1* signaling pathway is involved in EMT regulation and *TGF- β1* is the most representative member of *TGF- β1* protein family. So, we want to address the role of *TGF- β1* in migration and invasion in A549 cells. pCMV-*TGF- β1* and pCMV-vector control were transfected into A549 cells respectively. Then, we examined the expression levels of *N-cadherin*, *E-cadherin* and *Vimentin* in the transfected cells. The results showed that N-cadherin and Vimentin were expressed at a higher level in pCMV-*TGF- β1* group than in pCMV-vector control group by real-time PCR and western blotting, while E-cadherin was expressed at a higher level in pCMV-vector group than in pCMV-CTEN control group (S1 Fig A-B). Then, the *in-viro* scratch-wound assay in A549 cells showed that *TGF- β1* overepressed group migrated faster than vector control group (S1 Fig C). Further, transwell assay demonstrated that overexpression of *TGF- β1* promoted the invasion ability of A549 cells (S1 Fig D). Together, these results demonstrate that *TGF- β1* plays a positive role in EMT, cell migration and invasion of A549 cells.
(DOC)
