# Supplementary material for: CTEN induces epithelial-mesenchymal transition (EMT) and metastasis in non small cell lung cancer cells
Source: PLoS One. 2018 Jul 9;13(7):e0198823. doi: 10.1371/journal.pone.0198823 (PMC6037349; doi:10.1371/journal.pone.0198823)
Supplement: S1 Fig — TGF- β1 signaling pathway is involved in EMT regulation and TGF- β1 is the most representative member of TGF- β1 protein family. So, we want to address the role of TGF- β1 in migration and invasion in A549 cells. pCMV-TGF- β1 and pCMV-vector control were transfected into A549 cells respectively. Then, we examined the expression levels of N-cadherin, E-cadherin and Vimentin in the transfected cells. The results showed that N-cadherin and Vimentin were expressed at a higher level in pCMV-TGF- β1 group than in pCMV-vector control group by real-time PCR and western blotting, while E-cadherin was expressed at a higher level in pCMV-vector group than in pCMV-CTEN control group (S1 Fig A-B). Then, the in-viro scratch-wound assay in A549 cells showed that TGF- β1 overepressed group migrated faster than vector control group (S1 Fig C). Further, transwell assay demonstrated that overexpression of TGF- β1 promoted the invasion ability of A549 cells (S1 Fig D). Together, these results demonstrate that TGF- β1 plays a positive role in EMT, cell migration and invasion of A549 cells. (DOC) [file pone.0198823.s001.doc]

**Supporting Information**

*TGF-β1* promotes EMT, migration and invasion of human lung adenocarcinoma A549 cells

**
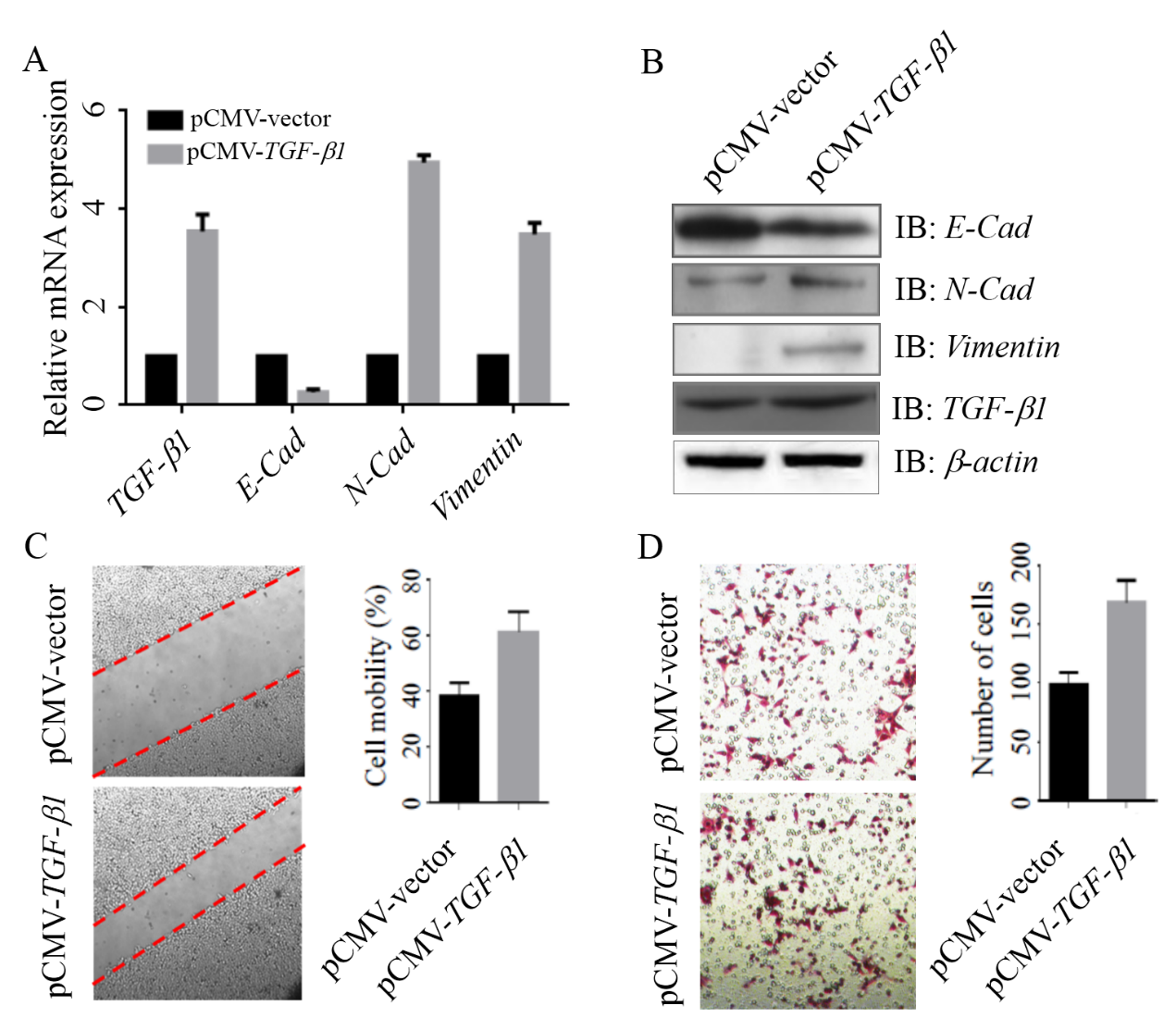
**

pCMV-*TGF-β1* and pCMV-vector control were transfected into A549 cells respectively. Expression level of *TGF-β1*, *E-cadherin (E-cad)*, *N-cadherin (N-Cad)* and *Vimentin* was evaluated by real-time PCR (A) and western blotting (B). (C) and (D) Representative images depicting the effect of *TGF-β1* overexpression on A549 cells migration (C) and invasion (D).
